# REST is a crucial regulator for acquiring EMT-like and stemness phenotypes in hormone-refractory prostate cancer

**DOI:** 10.1038/srep42795

**Published:** 2017-03-03

**Authors:** Yi-Ting Chang, Tzu-Ping Lin, Mel Campbell, Chin-Chen Pan, Shu-Hui Lee, Hsin-Chen Lee, Muh-Hwa Yang, Hsing-Jien Kung, Pei-Ching Chang

**Affiliations:** 1 Institute of Microbiology and Immunology, National Yang-Ming University, Taipei, Taiwan; 2 Institute of Clinical Medicine, National Yang Ming University, Taipei, Taiwan; 3 Department of Urology, School of Medicine, and Shu-Tien Urological Research Center, National Yang-Ming University, Taipei, Taiwan; 4 Department of Urology, Taipei Veterans General Hospital, Taipei, Taiwan; 5 UC Davis Comprehensive Cancer Center, University of California, Davis, CA 95616, USA; 6 Department of Pathology, Taipei Veterans General Hospital, National Yang-Ming University, Taipei, Taiwan; 7 Institute of Pharmacology, National Yang-Ming University, Taipei, Taiwan; 8 Institute of Clinical Medicine, National Yang-Ming University, Taipei 11221, Taiwan; 9 Institute of Biotechnology in Medicine, National Yang-Ming University, Taipei 11221, Taiwan; 10 Division of Hematology and Oncology, Department of Medicine, Taipei Veterans General Hospital, Taipei 11217, Taiwan; 11 Genomics Research Center, Academia Sinica, Taipei 11529, Taiwan; 12 Immunity and Inflammation Research Center, National Yang-Ming University, Taipei 11221, Taiwan; 13 Genome Research Center, National Yang-Ming University, Taipei, Taiwan; 14 Division of Molecular and Genomic Medicine, National Health Research Institutes, 35 Keyan Road, Zhunan, Miaoli County 35053, Taiwan; 15 Department of Biochemistry and Molecular Medicine, University of California, Davis, CA 95616, USA; 16 Institute for Translational Medicine, College of Medical Science and Technology, Taipei Medical University, 250 Wu-Xin Street, Taipei City, Taiwan; 17 Center for Infectious Disease and Cancer Research, Kaohsiung Medical University, Kaohsiung, Taiwan, R.O.C

## Abstract

Castration-resistance prostate cancer (CRPC), also known as hormone-refractory prostate cancer (HRPC), requires immediate attention since it is not only resistant to androgen ablation, chemo- and radiotherapy, but also highly metastatic. Increasing evidence suggests that enrichment of neuroendocrine (NE) cells is associated with CRPC. Here, combined RNA-seq and ChIP-seq analysis reveals that REST is involved in epithelial-mesenchymal transition (EMT) and stemness acquisition in NE differentiated prostate cancer (PCa) cells via direct transcriptional repression of Twist1 and CD44. Specifically we show that short-term knockdown of REST induces NE differentiation of LNCaP cells. Long-term REST knockdown enhanced the expression of Twist1 and CD44, cell migration and sphere formation. Overexpression of REST in hormone-refractory CWR22Rv1 PCa cells significantly reduces Twist1 and CD44 expression, cell migration and sphere formation. Collectively, our study uncovers REST in regulating EMT and stemness properties of NE PCa cells and suggests that REST is a potential therapeutic target for CRPC.

Prostate cancer (PCa) is a major cancer affecting older male population^1^. This disease is first manifested as an androgen-dependent cancer that can be successfully treated with androgen deprivation therapy (ADT). Unfortunately, an aggressive and ultimately fatal castration-resistance prostate cancer (CRPC) eventually develops a few years after successful treatment^2^. As the average lifespan increases due to the advances in treatment of chronic diseases, more elderly males will be living long enough to suffer the consequences of the relapse of PCa. Since CRPC is highly radio/chemoresistant and metastatic^2^,^3^, much interest has been focused on finding novel targeted therapies for this advanced type of PCa.

One fascinating but understudied feature of CRPC is its association with an increased number of neuroendocrine-like (NE-like) cells^3^, an androgen receptor (AR)-negative and non-proliferative terminally differentiated type of cell that expresses high levels of NE markers, including tubulin III, synaptophysin (SYP), and chromogranin A (CgA), and displays a neurite-like morphology^4^. NE-like cells are highly resistant to chemotherapy and release paracrines that promote the proliferation of neighboring cells^5^. Therefore, NE-like cells have long been considered as one of the major causes of difficulties in treating CRPC. Most interestingly, emerging evidence showing the association of NE and stem-like phenomena^6^ raises a new concept of regression of advanced NE PCa to stemness^7^. Cancer stem cells (CSCs) have long been considered to be crucial for tumor initiation, radio/chemoresistance and metastasis^8^. Acquisition of stemness by NE-like cells provides a novel insight for the aggressiveness of CRPC.

In addition to androgen deprivation^3^,^9^, hypoxia^10^,^11^ and IL-6 treatment^12^,^13^ have also been shown to induce neuroendocrine differentiation (NED) of PCa cells *in vitro*. NE-like cell development relies on a network of transcriptional reprogramming that controls the acquisition and maintenance of neuronal features. Recent reports including ours provide evidence showing that repressor element-1 (RE-1) silencing transcription factor (REST) expression is significantly reduced in relapsed PCa tissue^9^,^12^ and reduction of REST is responsible for NED of PCa cells^9^,^10^,^11^,^12^,^13^. REST was originally identified as a transcription repressor for neuron-specific genes in neuronal progenitor and non-neuronal cells^14^. Although REST was initially identified as a master negative regulator of neurogenesis, recent studies revealed its unanticipated roles in tumorigenesis and stem cell self-renewal. The paradoxical character of REST in tumor suppression and stimulation of oncogenesis is now recognized that, in general, REST functions as a tumor suppressor in epithelial cells that express REST but plays an oncogenic role in neuronal cells that normally do not express REST (reviewed in ref. 15). The oncogenic function of REST was identified in medulloblastoma^16^,^17^ and glioblastoma^18^, while the tumor suppressor role of REST was elucidated in colorectal cancer^19^,^20^, breast cancer^21^,^22^, PCa^9^,^10^,^11^,^12^,^13^ and small cell lung cancer (SCLC)^23^,^24^. Interestingly, many of these REST-deficient tumors acquire a NE phenotype exhibiting aberrant expression of neuronal genes^9^,^10^,^11^,^12^,^13^,^24^,^25^. In addition to tumorigenesis, contradictory findings of REST in maintaining pluripotency and self-renewal of ESCs were also reported^26^,^27^,^28^,^29^. One recent report provided an explanation for this contradiction by suggesting a passage-dependent response, as REST deficiency impaired pluripotency of ESCs in early passage and restoration of impaired self-renewal upon prolonged culture^30^. In PCa, REST was found to be a mediator of AR^9^ and induced NED upon downregulation^10^,^11^,^12^. However, whether a prolonged decrease of REST results in NE-like cells acquiring stemness is largely unknown. As stemness features are the root cause of chemoresistance and aggressiveness, identification of the molecules that drive this process will provide novel therapeutic interventions.

To determine the potential of downregulating REST for NE-like cells to acquire stemness, we first performed a Gene Ontology (GO) pathway analysis of RNA-seq data of genes that are upregulated by REST knockdown or IL-6 treatment. Interestingly, other than IL-6 signaling, stemness, epithelial-mesenchymal transition (EMT) and cancer pathways were also identified. Consistent results were observed in gene set enrichment analysis (GSEA). RNA-seq analyses show that (i) short-term REST knockdown induces only NED, (ii) long-term REST knockdown induces EMT and stemness in NE differentiated LNCaP cells and (iii) both short- and long-term REST overexpression repress EMT and stemness of CWR22Rv1 cells, a castration-resistant PCa cell line. Interestingly, we also determined that REST is the transcription repressor that directly targets and represses the mesenchymal marker Twist1 and a stemness marker CD44. Taken together, our findings identified REST as the first epigenetic modifier involved in promoting not only NED but also EMT and stemness in advanced PCa upon its downregulation.

## Materials and Methods

### Cell culture and plasmids

LNCaP, CWR22Rv1, PC3, and DU145 cells were cultured in RPMI 1640 (Gibco/Invitrogen, 31800–014) supplemented with 10% fetal bovine serum (FBS) (Hyclone, SH30071.03), 1X penicillin/streptomycin (Invitrogen 1514-0122) and 0.3 mg/ml L-glutamine (Sigma-Aldrich, G8540). LNCaP-TR-shREST cells^12^ were maintained as described for LNCaP with addition of 5 μg/ml of blasticidin S (InvivoGen, ant-bl-1) and 50 μg/ml of zeocin (InvivoGen, ant-zn-1). For REST-inducible CWR22Rv1 cells, REST cDNA was introduced into the pLenti4-CMV/TO vector and introduced into CWR22Rv1-TR cells by lentiviral transduction. After selection with 200 μg/ml zeocin induction efficiency was tested. CWR22Rv1-TR-REST were maintained as described for CWR22Rv1 and supplemented with 5 μg/ml of blasticidin S and 100 μg/ml of zeocin. The pLKO.1, pLKO.1-shTwist1, and pLKO.1-shCD44 vectors (Academia Sinica, RNAi Core) were used to generate control lentivirus or lentiviral particles for knockdown Twist1 and CD44 expression.

### RNA-seq and gene ontology (GO) analysis

Total RNA harvested from 100 ng/ml IL-6-treated LNCaP cells was analyzed by high-throughput sequencing at the Sequencing Core at National Yang-Ming University VYM Genome Research Center. Sequencing reads were trimmed by Bowtie and aligned to hg19 by TopHat. Cufflinks was used to calculate the transcript abundances. Transcriptome information was obtained from RefSeq Transcripts-2015-11-03. Ingenuity Pathway Analysis (IPA) (http://www.ingenuity.com) and gene set enrichment analysis (GSEA) (http://software.broadinstitute.org/gsea/index.jsp) were used to analyze the biological functions.

### Reverse transcription and quantitative PCR (RT-qPCR)

Total RNA was isolated by TRIzol reagent (Invitrogen, 15596-018) and cDNA was synthesized using Oligo-dT and SuperScriptIII First-Strand Synthesis System (Invitrogen, 18080-085) according to manufacturer’s protocol. The qPCR primer pairs were designed by PerlPrimer (http://perlprimer.sourceforge.net/). Primer sequences are shown in Table S1. Quantification the expression level of each gene was carried out in Bio-Rad CFX96 Real-Time PCR Detection System. All genes were normalized against GAPDH.

### Immunoblotting and antibodies

Total cells lysates (TCLs) were prepared in RIPA buffer containing fresh protease inhibitor (Roche, 04693132001). TCLs were resolved on SDS-polyacrylamide gels, transferred to PVDF membrane, blocked with 5% BSA/TBST, hybridized with specific primary antibodies followed with appropriate HRP-conjugated secondary antibody, visualized by Pierce ECL Western Blotting Substrate (Thermo Scientific, 34080) and imaged via a Luminescence/Fluorescence Imaging System (FUJIFILM, LAS-4000). Primary antibodies were anti-REST (Millipore, 07–579), Tubulin III (Sigma Aldrich, T2200), SYP (GTX100865), Twist (GTX213110-01), CD44 (GTX102111), N-cadherin (Cell signaling, 4061), ZO-1 (Cell signaling, 8193), E-cadherin (Cell signaling, 3195), and GAPDH (GTX 100118).

### Immunofluorescence assay (IFA)

Cells were seeded on poly-L-lysine-coated coverslips (Marienfeld, 0111530). After treatment, cells were fixed by 4% paraformaldehyde/PBS, permeabilized, blocked with 1% BSA/PBS, stained at 4 °C with anti-CD44 (Thermo Scientific, MA5-13890) followed by fluorescent-tagged secondary antibody. After washing, nuclei were counterstained with Hoechst 33342 (Thermo Scientific Pierce 62249), mounted by mounting solution, and visualized using a fluorescence inverted microscope (Lecia, DMI4000B).

### Immunohistochemistry staining (IHC-staining)

Paraffin-embedded specimens from patients with primary, hormone-refractory, and bone metastatic PCa were collected at Taipei Veterans General Hospital (TVGH). Ethics was approved by the TVGH Review Board. Tissue sections were stained and visualized as described previously^12^ with anti-CgA antibody (Thermo Scientific, MA5-13096) and anti-REST antibody (BETHYL, IHC-00141). The histology and staining was evaluated by Dr. Chin-Chen Pan in a blind fashion.

### Flow cytometry analysis

Cells collected by trypsin treatment were washed with PBS, stained in 100 μl PBS containing anti-CD44 antibody (BioLegend, 103006) for 15 minutes on ice and washed again with PBS. Flow cytometry analysis was performed on BD FACSCalibur™ (BD Biosciences) and analyzed using FlowJo v10.1r5.

### Transwell migration and spheriod formation assay

For migration assay, cells were seeded in 24-well Thin-Cert^TM^ chambers with 8 μm-pore size (Greiner bio-one (662–638)). After three days, the cells were fixed and stained by Hoechst 33342. The migrated cells were imaged and counted in 10 microscopic fields. The image were quantified and averaged by using MetaMorph (Molecular Devices).

For sphere formation assays, cells were grown in suspension culture using serum-free RPMI supplemented with B27 (Invitrogen), 20 ng/ml EGF (ProSpec CYT-218), and 10ng/ml FGF-b (ProSpec CYT-218). Sphere diameter was measured using MetaMorph (Molecular Devices).

### Chromatin immunoprecipitation-sequencing (ChIP-Seq) and real-time qPCR

ChIP assays were performed following the protocol described by Farnham laboratory (provided at http://genomics.ucdavis.edu/farnham). ChIP grade rabbit polyclonal antibodies specific against REST (Millipore, 17–641), H3K4me1 (Abcam, ab8895) and H3ac (Millipore, 06–599), as well as rabbit non-immune serum IgG (Alpha Diagnostic International) were used. ChIP DNA was prepared from 4 × 10^8^ LNCaP cells. ChIP-seq library was prepared following the protocol from Illumina and subjected to sequencing. ChIP-Seq data was aligned onto hg19. The Partek software (Partek Genomics suite 6.6) was used to localize potential REST binding sites. Binding sites were verified by SYBR^®^ Green Based qPCR using a Bio-Rad CFX96 Real-Time PCR detection system. Specific primer sets were designed around the identified binding sites and listed in Table S1.

## Results

### Epithelial mesenchymal transition (EMT) and cell stemness are common pathways targeted by both IL-6 and REST

**Figure 1:**
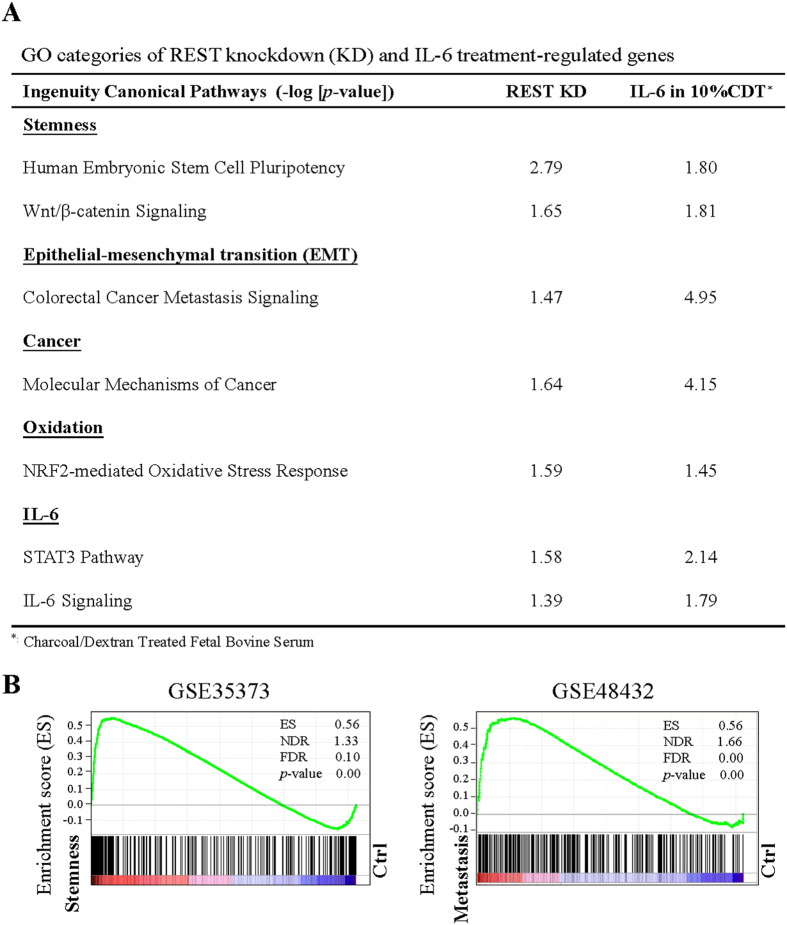
REST knockdown and IL-6 treatment share common regulatory pathways in NE differentiated PCa LNCaP cells. (**A**) Ingenuity canonical pathway (IPA) analysis of REST knockdown and IL-6 regulated genes. −log (*p value*) > 1.3 was considered statistically significant. (**B**) The GSEA result showing the correlation of REST knockdown and IL-6 co-regulated genes with PCa stemness (left panel) and metastasis (right panel).

Several laboratories including ours have demonstrated that REST is a novel regulator of human PCa cell NED^9^,^10^,^11^,^12^,^13^. The terminally differentiated NE cells are recognized as a potential cause of CRPC and apparent acquisition of stemness properties in advanced CRPC^7^. In light of a recent report showing the REST deficiency impairs pluripotency in early stage passage but restoring self-renewal properties upon prolonged culture^30^, we investigated signaling pathways, other than NED, that are regulated during IL-6 treatment and when REST protein levels are down modulated using Ingenuity Pathway Analysis (IPA) software. GO pathway analysis of genes regulated by IL-6 under androgen deprivation conditions (10% charcoal/dextran-treated FBS (CDT)) and REST knockdown showed that the 3735 and 4816 genes regulated by IL-6 treatment and REST knockdown, respectively, were commonly significantly enriched (−log [*P*-value] > 1.3) in IL-6, EMT, stemness, cancer and oxidation pathways. The identification of IL-6 pathway in REST knockdown cells is consistent with our previous report which showed REST as a potential downstream negative regulator targeted by IL-6^12^. In addition, oxidative stress and cancer-related signaling pathways were also observed. This suggests a role of downregulating REST in mediating IL-6-induced tumorigenesis in PCa. The most important finding in GO pathway analysis was the concomitant enrichment of EMT and stemness signaling in both IL-6 treatment and REST knockdown (Fig. 1A). To further identify common gene networks between the IL-6 and REST downregulation co-regulated genes and PCa stemness and metastasis, we ran a gene set enrichment analysis (GSEA) using microarray datasets from two separate GEO databases: (1) GSE35373: PCa stromal cells versus iPS cells induced from PCa stromal cells; and (2) GSE48432: LNCaP cell versus LNCaP cells overexpressing RANKL, a gene responsible for bone metastasis of cancer cells^31^. Consistent with our GO pathway analysis, GSEA revealed that the expression of the 497 genes co-regulated by IL-6 and REST downregulation was highly correlated with stemness (GSE35373) (Fig. 1B, left panel) and metastasis (GSE48432) (Fig. 1B, right panel). Taken together, these results suggest that REST may be involved in the regulation of EMT and stemness-related genes.

### NED of PCa cells correlates with EMT and CSC properties

**Figure 2:**
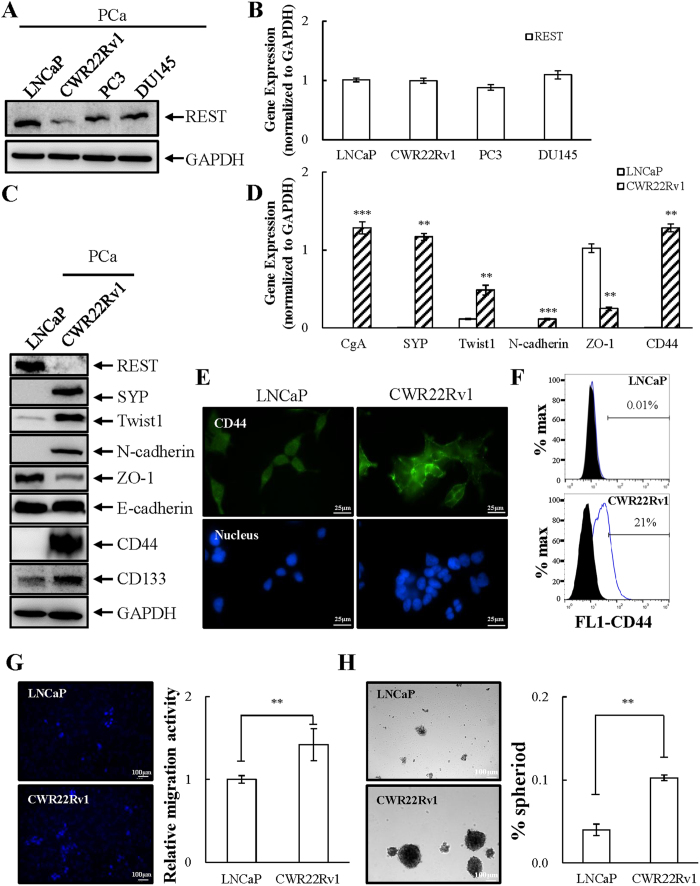
PCa cell lines profiled for REST expression and characterization of EMT and stemness signature. (**A** and **B**) LNCaP, CWR22Rv1, PC3, and DU145 were examined for REST protein by immunoblotting (**A**) and REST mRNA by RT-qPCR (**B**). n = 3. (**C**) Immunoblotting of protein expression for REST, NE marker SYP, and EMT and stemness markers as indicated in LNCaP and CWR22Rv1 cells. n = 3. (**D**) mRNA levels of NE and EMT markers and CD44 in LNCaP and CWR22Rv1 cells. n = 2. (**E** and **F**) Immunofluorescence micrographs (**E**) and flow cytometric analysis (**F**) of CD44 expression in LNCaP and CWR22Rv1 cells. n = 2. (**G** and **H**) LNCaP and CWR22Rv1 cells were seeded in transwell (1 × 10^4^) or cultured in defined serum-free sphere medium with growth factors (2 × 10^4^) and measured at 3 and 14 days, respectively. The number of migrated cells was quantified by the average from 5 microscopic fields (**G**). The number of spheres was calculated (**H**). Representative images (left) and quantification (right) in both PCa cell lines. n = 3. Data represent means ± SD. **p < 0.01, ***p < 0.001 by *Student’s-t* test.

Since REST appears to be a negative regulator of EMT and stemness of NE PCa cells, we investigated the effect of REST on the migration and sphere formation of PCa cells. First, we screened the expression level of REST in four PCa cell lines: LNCaP, CWR22Rv1, PC3, and DU145. LNCaP is an androgen-sensitive human prostate adenocarcinoma cell line with little NE characteristics^32^,^33^,^34^. CWR22Rv1 was derived from the relapsed CWR22 xenograft after castration displaying NE phenotype^35^. PC3 and DU145 are androgen receptor negative human prostate adenocarcinoma cell lines with some NE features^33^,^34^ but can be further induced to acquire additional NED phenotypes^36^. Consistent with previous results showing that REST is regulated by β-TrCP at protein level during NED^9^,^10^, the protein but not mRNA level of REST was significantly lower in CWR22Rv1 than that in the other three cell lines (Fig. 2A,B). We therefore selected REST-high LNCaP and REST-low CWR22Rv1 respectively to generate an inducible REST knockdown cell line, LNCaP-TR-shREST, and inducible REST overexpression, CWR22Rv1-TR-REST cells.

Following the pathway analysis, EMT and stemness markers were compared in LNCaP and CWR22Rv1 cells using immunoblotting. Expression of the epithelial marker ZO-1 but not E-cadherin was higher in LNCaP and the mesenchymal marker Twist1 and N-cadherin were higher in CWR22Rv1 (Fig. 2C). Consistently, transcription of ZO-1 was significantly lower and Twist1 and N-cadherin was higher in CWR22Rv1 (Fig. 2D). The expression of PCa stem cell-associated markers CD44 and CD133 were also analyzed^37^,^38^. Protein levels of both CD44 and CD133 were significantly higher in CWR22Rv1 (Fig. 2C). Immunofluorescence and cell surface flow cytometry analysis also showed that CD44 expression is higher in CWR22Rv1 cells (Fig. 2E,F). As EMT promotes cell migration and CD44 allows outgrowth of cells into spheres, cell migration and sphere formation ability between LNCaP and CWR22Rv1 cells were compared. To study this, LNCaP and CWR22Rv1 were seeded on the same day and the cell migration and sphere formation were measured after three and fourteen days, respectively. Consistent with expression profiles, cell migration (Fig. 2G) and sphere formation (Fig. 2H) were significantly higher in CWR22Rv1 cells. Altogether, we suggest that NE PCa cells may indeed share stemness and neuronal features.

### REST downregulation induces EMT and stemness in PCa LNCaP cells

**Figure 3:**
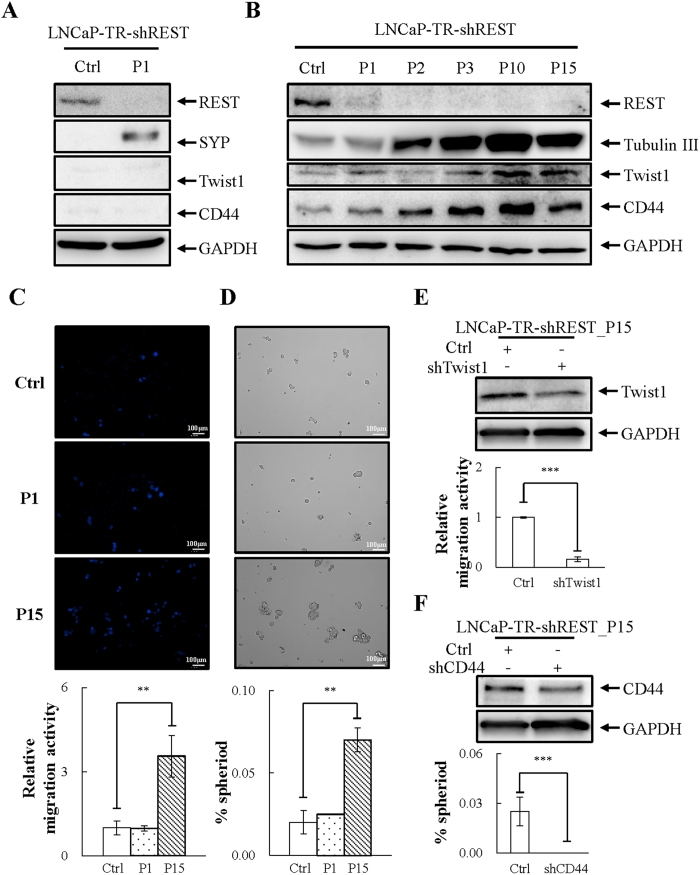
Long-term but not short-term REST knockdown promotes EMT and stemness properties of PCa LNCaP cells. (**A**) Immunoblotting of REST, NE marker synaptophysin (SYP), mesenchymal marker Twist1 and stemness marker CD44 in LNCaP-TR-shREST cells receiving 1 μg/ml Dox for 3 days (P1) compared to no Dox control. n = 3. (**B**) Total cell lysates (TCLs) were prepared from LNCaP-TR-shREST treated as described in (**A**) for 3 (P1), 6 (P2), 9 (P3), 30 (P10), and 45 (P15) days and then immunoblotted with antibodies as indicated. n = 3. (**C**) LNCaP-TR-shREST cells were treated as described in (**A**) for 45 (P15) days, seeded on the transwell membrane, and after three days the cells were stained with Hoechst. Representative images (top) and quantification (bottom) of migrated LNCaP cells. (**D**) LNCaP-TR-shREST cells were treated as described in (**C**) and then cultured in defined serum-free sphere medium for 14 days. Representative images (top) and quantification (bottom) of sphere formation in LNCaP cells. (**E**) Immunoblotting showed the successful knockdown of Twist1 in LNCaP-TR-shREST cells receiving 1 μg/ml Dox for 45 days (P15). Representative images (top) and quantification (bottom) of cell migration in LNCaP cells described in (**E**). (**F**) Immunoblotting showed the successful knockdown of CD44 in LNCaP-TR-shREST cells receiving 1 μg/ml Dox for 45 days (P15). Representative images (top) and quantification (bottom) of sphere formation in LNCaP cells described in (**F**). n = 2. Data represent means ± SD. **p < 0.01, ***p < 0.001 by *Student’s-t* test.

Since low expression level of REST is correlated with high migration and sphere formation, we further investigated whether knockdown REST also contributes to induction of EMT and cell stemness. Consistent with our previous report, knockdown of REST for 3 days induced NED, as demonstrated by an increase of SYP (Fig. 3A). However, the knockdown of REST for 3 days did not increase the expression of CD44 and Twist1 (Fig. 3A) nor affect cell migration or sphere formation (Fig. 3C,D). Among the studies of NED of PCa cells, there is evidence which demonstrates different phenotypes of LNCaP cells following long-term treatment with inducers of NED^39^,^40^. Moreover, the restoration of self-renewal character of REST-deficient ESCs upon prolonged culture^30^ prompted us to hypothesize that REST knockdown initially induced NED in LNCaP cells followed by the acquisition of stemness properties. This suggests that induction of EMT and stemness by REST downregulation may require a prolonged treatment. To elucidate this, LNCaP-TR-shREST cells were treated with 1 μg/ml doxycycline (Dox) continuously for up to 15 passages. Each passage indicates a Dox treatment for 3 days. Cells of passage 1, 2, 3, 10 and 15 (P1, P2, P3, P10 and P15) were collected for immunoblotting. As shown in Fig. 3B, NED was induced in early passage (P1, P2 and P3) and the increase of Twist1 and CD44 protein was detected in late passage (P10 and P15). Following the expression result, the control, P1 and P15 cultures of REST knockdown LNCaP cells were seeded on the same day for migration and sphere formation assays. Consistently, long-term knockdown of REST in LNCaP increased cell migration (Fig. 3C) and sphere formation (Fig. 3D) ability. The increase in sphere formation by REST knockdown was also identified after one passage (Fig. S1).

To test whether upregulation of Twist1 and CD44 is indeed required for REST knockdown-induced migration and sphere formation, Twist1 and CD44 were individually transiently knocked down in the P15 of REST knockdown LNCaP cells. The successful knockdown of Twist1 and CD44 were first confirmed by immunoblotting (Fig. 3E,F). Knockdown Twist1 and CD44 significantly inhibited cell migration (Fig. 3E) and sphere formation (Fig. 3F), respectively. Altogether, these data suggest that upregulation of Twist1 and CD44 is a prerequisite for REST knockdown-induced EMT and stemness NE PCa LNCaP cells acquiring properties.

### REST overexpression inhibits EMT and stemness in PCa CWR22Rv1 cells

**Figure 4:**
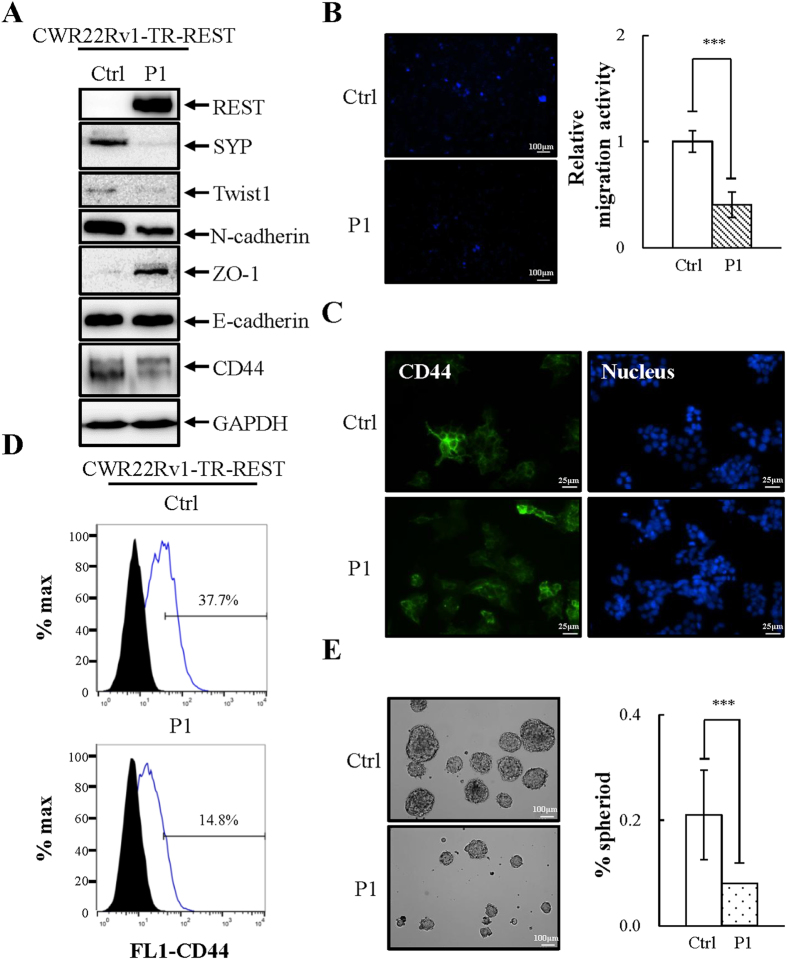
Short-term overexpression of REST inhibits EMT and stemness properties of CWR22Rv1 cells. (**A**) Immunoblotting of REST, NE marker SYP, and EMT and stemness markers in CWR22Rv1-TR-REST cells receiving 1 μg/ml Dox for 3 days (P1) compared to no Dox control. n = 3. (**B**) CWR22Rv1-TR-REST cells treated as described in (**A**) were seeded on the transwell membrane and after three days the cells were stained with Hoechst. Representative images (left) and quantification (right) of migrated CWR22Rv1 cells. n = 3. (**C** and **D**) Immunofluorescent micrographs (**C**) and flow cytometric analysis (**D**) of CD44 expression in CWR22Rv1 cells treated as described in (**A**). n = 2. (**E**) CWR22Rv1-TR-REST cells treated as described in (**A**) were then cultured in defined serum-free sphere medium for 14 days. Representative images (left) and quantification (right) of sphere formation in CWR22Rv1 cells. n = 3. Data represent means ± SD. ***p < 0.001 by *Student’s-t* test.

**Figure 5:**
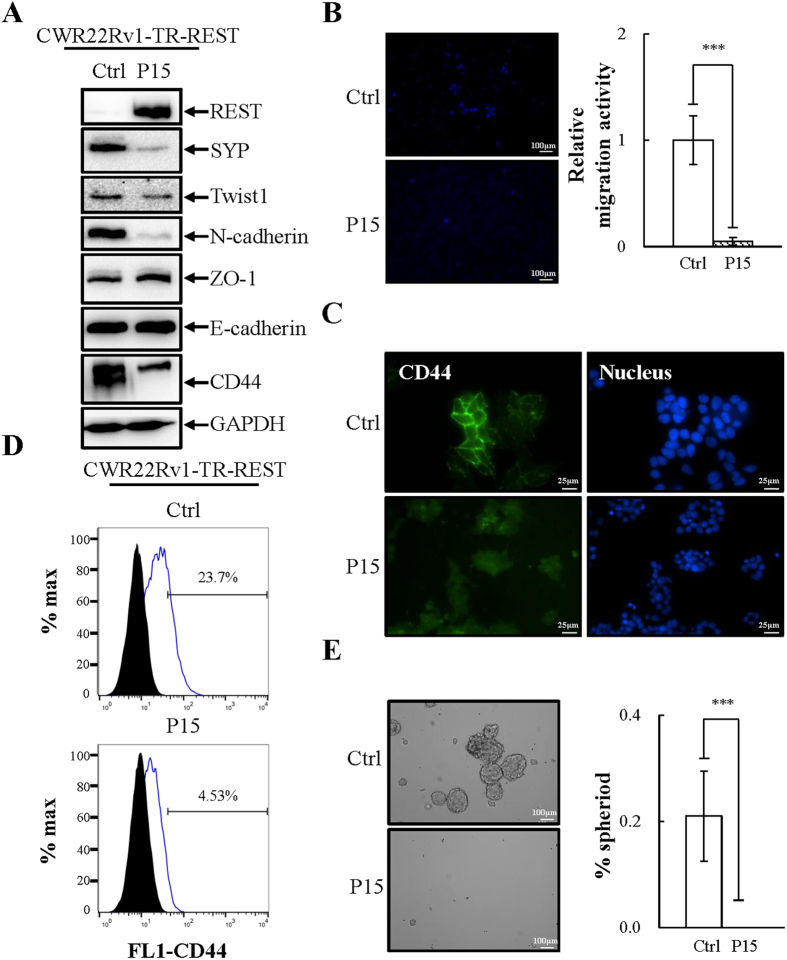
Long-term overexpression of REST inhibits EMT and stemness properties of CWR22Rv1 cells. (**A**) Immunoblotting of REST, NE marker SYP, and EMT and stemness markers in CWR22Rv1-TR-REST cells receiving 1 μg/ml Dox for 45 days (P15) compared to no Dox control. n = 2. (**B**) CWR22Rv1-TR-REST cells treated as described in (**A**) were seeded on the transwell membrane and after three days the cells were stained with Hoechst. Representative images (left) and quantification (right) of migrated CWR22Rv1 cells. n = 3. (**C** and **D**) Immunofluorescent micrographs (**C**) and flow cytometric analysis (**D**) of CD44 expression in CWR22Rv1 cells treated as described in (**A**). n = 2. (**E**) CWR22Rv1-TR-REST cells treated as described in (**A**) were then cultured in defined serum-free sphere medium for 14 days. Representative images (left) and quantification (right) of sphere formation in CWR22Rv1 cells. n = 3. Data represent means ± SD. ***p < 0.001 by *Student’s-t* test.

We next investigated whether overexpression of REST might inhibit the migration and sphere formation ability of CWR22Rv1. To this end, the control, P1 and P15 cultures of REST overexpressed CWR22Rv1-TR-REST cells were seeded on the same day and later collected to examine EMT and stemness characteristics (P1, Fig. 4; P15, Fig. 5). Interestingly, both short and long term Dox-induced ectopic expression REST resulted in reduction of the mesenchymal marker Twist1 and N-cadherin and increase of the epithelial marker ZO-1 (Figs 4A and 5A). Moreover, REST-overexpressing cells exhibited a decrease in migration compared with non-induced control (Figs 4B and 5B, and S2A). The changes of CD44 expression was analyzed using immunoblotting (Figs 4A and 5A), immunofluorescence (Figs 4C and 5C) and flow cytometry analysis (Figs 4D and 5D). In REST-expressing cells, expression of the stemness marker CD44 was significantly downregulated. REST overexpressing CWR22Rv1 cells also showed decreased stemness as measured by sphere formation assay (Figs 4E and 5E). The decrease in sphere formation by short-term and long-term REST overexpression was also identified after two passages (Fig. S2B,C). A decrease of mesenchymal and stemness markers and an increase of an epithelial marker indicates a loss of mesenchymal and stemness properties of CWR22Rv1 cells after restoration of REST expression.

### REST directly represses CD44 and Twist1 expression

**Figure 6:**
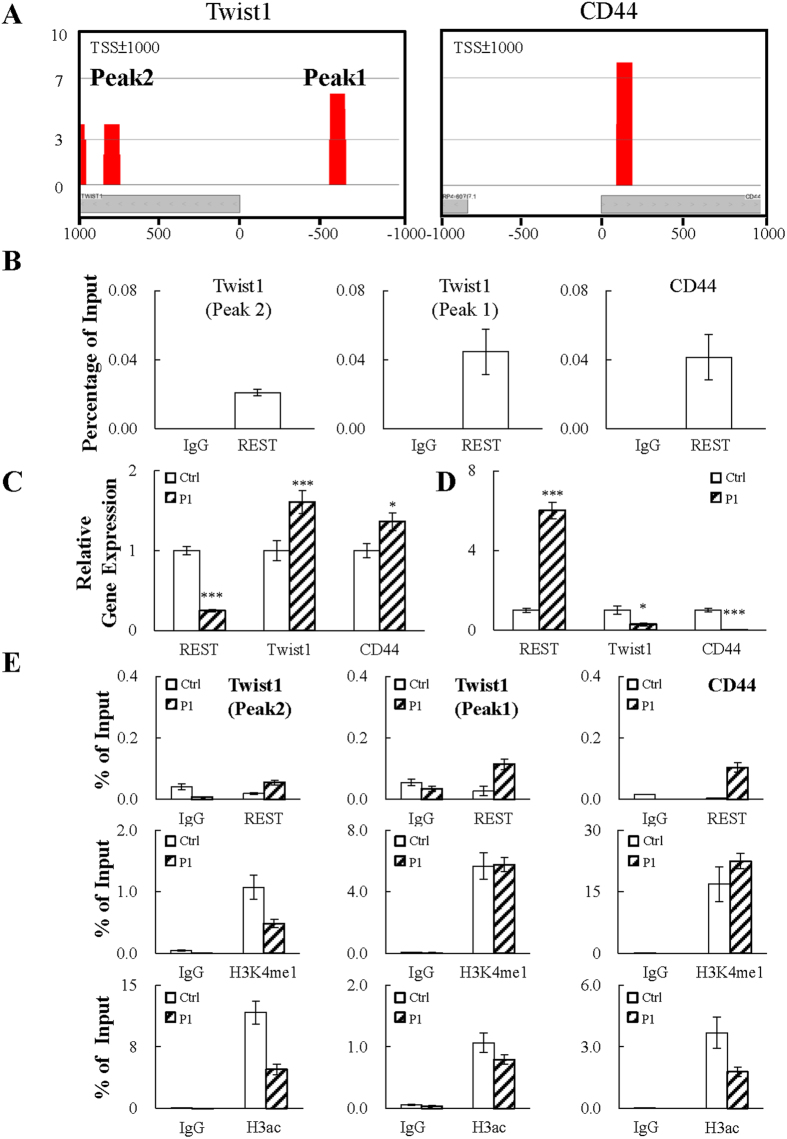
REST specifically binds and represses the promoters of CD44 and Twist1. (**A**) ChIP-seq for REST was performed using chromatin prepared from LNCaP cells. The rectangles indicate potential REST binding site(s) located on the Twist1 and CD44 promoter regions (TSS ± 1000 bp). (**B**) ChIP-qPCR assay confirmation of REST binding on Twist1 and CD44 promoters. The enrichment of each amplicon was normalized to input. n = 2. (**C** and **D**) RT-qPCR analysis of mRNA levels of REST, Twist1 and CD44 in REST knockdown LNCaP-TR-shREST (**C**) and REST overexpression CWR22Rv1-TR-REST (**D**) cells receiving 1 μg/ml doxycycline (Dox) (P1). Cells without Dox treatment (Ctrl) were used as control and arbitrarily set to 1. n = 2. (**E**) ChIP assays for REST, H3ac and H3K4me1 were performed using chromatin prepared from CWR22Rv1-TR-REST cells before (Ctrl) and after Dox treatment for three days (P1). The enrichment of each amplicon was quantified by qPCR and normalized to input. n = 2. Data represent means ± SD. *p < 0.05, ***p < 0.001 by *Student’s-t* test.

Since REST is best known as a transcription repressor, the transcription data prompted us to hypothesize that genes highly expressed in low REST containing CWR22Rv1 cells, such as Twist1, N-cadherin and CD44, may be potential direct targets of REST. To study this, we performed a chromatin immunoprecipitation-sequencing (ChIP-seq) in LNCaP cells using REST-specific antibody. As N-cadherin is the target gene of Twist1 in human breast and prostate epithelial cells^41^,^42^, Twist1 and CD44 were therefore chosen for further analysis. As shown in Fig. 6A, the ChIP-seq result revealed one and two REST binding sites on the promoter region (TSS+/− 1 Kb) of CD44 and Twist1, respectively. ChIP-seq data was further confirmed by real-time quantitative PCR (qPCR). Consistent with our ChIP-seq results, real-time qPCR data showed REST displayed a significant level of binding on CD44 and Twist1 promoters (Fig. 6B). Since direct binding of REST represses gene expression, both the REST inducible knockdown and overexpression cell lines were used for RT-qPCR analysis. Consistent with our ChIP data, knockdown REST in LNCaP cells resulted in an increase in CD44 and Twist1 expression (Fig. 6C) and, conversely, overexpression REST in CWR22Rv1 cells decreased their expression (Fig. 6D). Direct inhibition of CD44 and Twist1 promoter activity by REST was further confirmed by luciferase reporter assay. To this end, we cloned the promoter regions of CD44 and Twist1 with or without REST binding site(s) into a luciferase reporter plasmid (Fig. S3A). Consistently, the results showed that REST inhibited the luciferase activity driven by CD44 and Twist1 promoter more significantly than the corresponding REST binding site deficient mutants (Fig. S3B).

REST represses target gene expression by recruiting histone deacetylase and H3K4me2 histone demethylase to remove the active histone marks H3ac and H3K4me2, respectively. We conducted ChIP assays in CWR22Rv1-TR-REST cells using anti-REST, anti-H3K4me1 and anti-H3ac specific antibodies. The increase in REST binding to Twist1 and CD44 promoters was first confirmed. Consistent with our hypothesis, REST overexpression increased the levels of H3K4me1 on the CD44 promoter which represents the removal of H3K4me2 and decreased H3ac levels on the promoter (Fig. 6E; right panel). On the other hand, REST overexpression only decreased the levels of H3ac in both of REST binding sites on the Twist1 promoter (Fig. 6E; left and middle panel). The decreased H3K4me1 levels on one of the REST binding site on the Twist1 promoter suggests the completely removal of H3K4 methylation. These results indicate the REST-mediated CD44 and Twist1 silencing requires histone deacetylation and H3K4 demethylation and the potential of differential regulation of CD44 and Twist1 by REST.

### The association of CD44 and Twist1 expression and PCa relapse and bone metastasis

**Figure 7:**
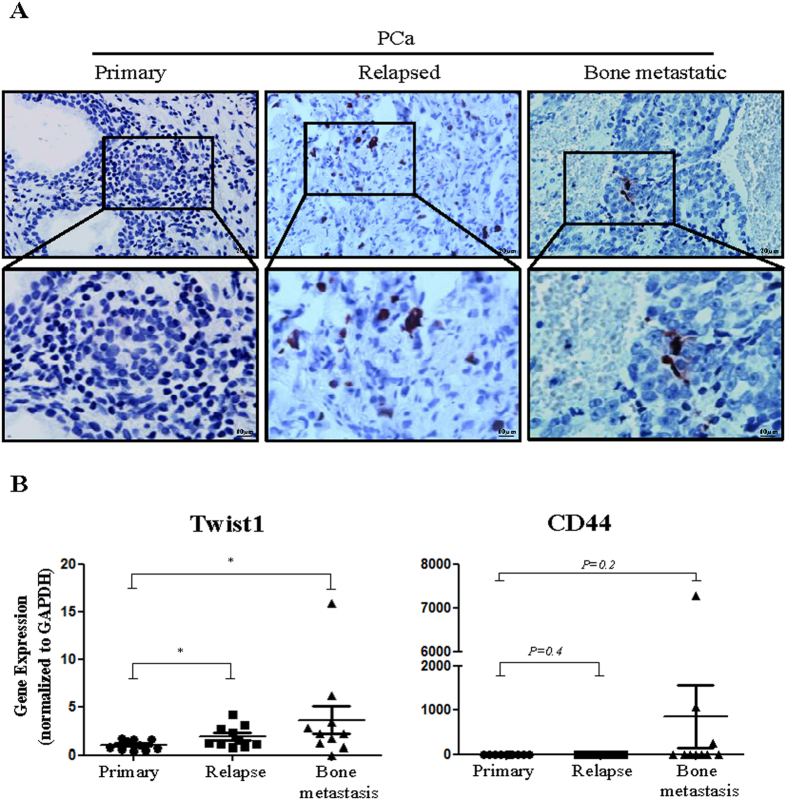
Analysis of human primary, relapsed and bone metastatic PCa specimens for the expression of CD44 and Twist1. (**A**) IHC staining was performed on ten primary, ten relapsed and ten bone metastatic PCa specimens using a specific antibody against a NE marker CgA. Representative images of CgA expression are shown depicting negative staining in primary and positive staining in relapsed and bone metastatic PCa. (**B**) The RNA level of CD44 and Twist1 were measured in each specimen by RT-qPCR. The statistical significance was calculated between the three groups of specimens using Student’s *t-test*. Statistical significance between groups is indicated. Data represent means ± SD. *p < 0.05 by *Student’s-t* test.

REST expression was found significantly reduced in relapsed PCa compared with tumors that had not relapsed^9^. Statistical analysis demonstrated that low REST expression is associated cancer recurrence^9^. Following the demonstration that Twist1 and CD44 are upregulated in NE differentiated PCa cells and can mediate EMT and cell stemness, we further analyze the expression of these two genes and REST in ten specimens from primary, relapsed, and bone metastatic PCa. As expected, NE marker CgA was detected (Fig. 7A) and REST was decreased (Fig. S4) in both the relapsed and bone metastatic PCa specimens. Expression of Twist1 was significantly increased in relapsed and bone metastatic PCa (Fig. 7B; left panel). Interestingly, the bone metastatic PCa expressed an even higher expression of Twist1. A trend in favor of upregulation of CD44 in bone metastatic PCa, but not relapsed PCa, relative to primary PCa was observed (Fig. 7B; right panel), even though statistical significance was not reached, possibly due to the low sample size (n = 10). Overall these results indicate the gradual acquisition of EMT in CRPC and cell stemness in bone metastatic PCa.

## Discussion

Here, we demonstrated that downregulation of REST is important for acquiring the EMT and CSC characteristics of terminal differentiated NE PCa cells by epigenetic derepression of Twist1 and CD44, providing a novel mechanism of REST-mediated epigenetic control of cell fate. Mechanistically, we found that for the transrepression of CD44, recruitment of REST to the promoter region reduces active histone marks H3K4me2 and H3ac (Fig. 6E; right panel). However, for Twist1, recruitment of REST only reduces H3ac (Fig. 6E; left and middle panel). These processes result in the acquisition of stemness and EMT properties in NE differentiated PCa cells and may explain the long-unanswered question related to high metastatic activity of advanced CRPC that exhibit NE features.

REST is a transcription repressor that is involved in maintaining pluripotency of neuron progenitor cells^14^ and ESCs^43^. REST regulates genes by direct binding to a 21 bp consensus sequence, named RE-1, in target promoter regions and recruits distinct corepressor complexes of Sin3/HDAC and CoREST/LSD1 through its N-terminal repressor domain (RD) 1 and C-terminal RD2, respectively, in a gene-specific manner^44^,^45^. Proper silencing of REST-repressed genes is essential for maintaining embryonic stem cells (ESCs) pluripotency and normal embryogenesis^43^. Downregulation of REST that consequently induced REST-targeted neuron differentiation-related genes and resulted in neurogenesis^46^. Here, we describe new functional genes, including Twist1 and CD44, can be targeted by REST (Fig. 6). We took advantage of two PCa cell lines: LNCaP that has little NE phenotype and spontaneously expresses a high level of REST, and CWR22Rv1, that exhibits a NE phenotype and expresses a very low level of REST (Fig. 2A,C).

Twist1 is a transcription factor that has a well characterized role in inducing EMT to promote tumor metastasis^41^,^47^. Twist1 induces EMT by enhancing the expression of the mesenchymal marker N-cadherin^42^ and suppressing the epithelial marker E-cadherin^48^. Here we showed that CWR22Rv1 expresses higher levels of Twist1 and N-cadherin (Fig. 2C,D). Though CWR22Rv1 cells do not have lower levels of E-cadherin when compared with LNCaP, lower expression of ZO-1, another epithelial marker, was identified (Fig. 2C,D). There is currently no evidence showing the direct targeting of ZO-1 by Twist1. However, emerging evidence showing that repression of ZO-1 expression in cancer cells during EMT involves transcription factors including Twist1^49^,^50^. Consistently, our REST overexpression data showed that overexpression of REST in CWR22Rv1 cells significantly reduced Twist1 and N-cadherin expression, along with increased ZO-1 level (Figs 4A and 5A). Our REST knockdown experiments in LNCaP cells further support the notion that REST downregulation not only induces NED of PCa cells but also drives NE differentiated PCa cells dedifferentiation to an EMT-like phenotype, as reflected by (i) increased Twist1 expression (Fig. 3B) and (ii) inhibition of cell migration by Twist1 knockdown in REST downregulation induced NE differentiated PCa cells (Fig. 3E).

CD44 is an important marker for various CSCs and has been used to isolate prostate CSCs^37^,^51^. In this study, we found that CWR22Rv1 cells express high levels of CD44 compared to PCa LNCaP cells (Fig. 2C–F). Overexpression REST in CWR22Rv1 significantly reduced CD44 (Figs 4A,C,D and 5A,C,D) and inhibited the sphere formation ability of CWR22Rv1 (Figs 4E and 5E). Consistently, knockdown CD44 significantly reduced REST downregulation induced sphere formation of PCa LNCaP cells (Fig. 3F). Together, our data suggest that downregulation of REST mediated dedifferentiation of NE PCa cells towards EMT and CSCs. Since EMT and CD44 are both highly associated with metastasis and drug resistance, our finding here may be the potential novel mechanisms for the highly metastatic and chemoresistant properties of CRPC, an advanced PCa exhibiting NE features.

One interesting, but not answered, question is how a transcriptional repressor targets and represses both transdifferentiation and pluripotency-promoting genes. Our hypothesis is the differential DNA binding mechanism of REST results in different binding affinities and kinetics on different promoters. It is well known that REST binds to genomic sequences that contain canonical or non-canonical RE-1 sites. However, a genome-wide profiling of REST binding and associated histone modifications showed similar outcomes of REST binding to canonical and non-canonical RE-1 sites^52^. This suggests that mechanisms other than direct recognition of RE-1 sites exist. One fascinating finding in recent years is the identification of long non-coding RNA (lncRNAs) as regulators for transcription and their involvement in pathogenesis especially in cancer^53^. Nuclear lncRNAs interact with and guide epigenetic regulatory proteins to target loci, which results in transcriptional alterations^54^. Proteins involved in lncRNA-mediated epigenetic regulation of gene expression include DNA-methyltransferase 3 A (DNMT3A), Polycomb repressive complex 2 (PRC2), REST, and CoREST (reviewed in ref. 55). This prompts us to hypothesize that lncRNA-mediated recruitment of REST protein is another potential mechanism for REST-mediated transcription regulation. This hypothesis is supported by the finding that there is no canonical and non-canonical RE-1 site identified by JASPAR (http://jaspar.genereg.net/) in the promoters region of Twist1 and CD44. Moreover, significantly more aligned reads were identified in RE-1 sites on promoters region of SYP and CgA (Fig. S5) compared with the REST enrichment peaks identified by ChIP-seq in Twist1 and CD44 promoter (Fig. S1). This perhaps suggests that the RNA secondary structure of an associated lncRNA may hinder the antibody from binding to REST during ChIP-assay. However, it should be pointed out that further studies need to be done to identify the lncRNAs and prove this hypothesis.

Differential kinetic regulation of transcriptional derepression between NE markers and Twist1 and CD44 was also identified during REST knockdown. As shown in Fig. 3B, derepression of Twist1 and CD44 by REST knockdown is significantly delayed to up to 10 passages. Following our hypothesis, this suggests that lncRNA-associated REST protein is more stable in both protein stability and chromatin binding. Therefore, the promoters of Twist1 and CD44 are still inhibited by REST even when REST protein level is below the detection level of immunoblotting. The REST protein is tightly regulated by the E3 ubiquitin ligase β-TrCP through the proteasomal degradation pathway^56^. Phosphorylation of serine residues within non-canonical degron motifs in REST C-terminal enables binding by β-TrCP and priming for ubiquitin-dependent proteasome-mediated degradation^57^. To prevent β-TrCP-mediated degradation, lncRNAs associated with REST may hinder either the phosphorylation or ubiquitination sites of REST and prevent it from modification and degradation. Testing this hypothesis is a very interesting topic for future studies. However, intensive experiments are needed to answer this question.

In summary, we identified Twist1 and CD44 as novel REST targeted genes. Our findings provide new insight into the epigenetic regulation of Twist1 and CD44 by REST and consequently reveal the potential role of REST in helping NE differentiated PCa cells acquire EMT and CSC phenotypes. NE differentiated PCa cells have long been proposed as the root cause of CRPC. Recently, Drs. Ellis and Loda suggested that those terminally differentiated NE PCa cells may regress to stemness in advanced CRPC^7^. This is the first report providing evidence to support this concept. If our hypothesis is correct, stabilization of REST may serve as a specific target in aggressive CRPC therapy.

## Additional Information

**How to cite this article**: Chang, Y.-T. *et al*. REST is a crucial regulator for acquiring EMT-like and stemness phenotypes in hormone-refractory prostate cancer. *Sci. Rep.* **7**, 42795; doi: 10.1038/srep42795 (2017).

**Publisher's note:** Springer Nature remains neutral with regard to jurisdictional claims in published maps and institutional affiliations.

## Supplementary Material

Supplementary Information
