# Supplementary material for: REST is a crucial regulator for acquiring EMT-like and stemness phenotypes in hormone-refractory prostate cancer
Source: Sci Rep. 2017 Mar 3;7:42795. doi: 10.1038/srep42795 (PMC5335619; doi:10.1038/srep42795)
Supplement: Supplementary Information [file srep42795-s1.pdf]

REST is a crucial regulator for acquiring EMT-like and stemness phenotypes in hormone-refractory prostate cancer

Yi-Ting Chang, Tzu-Ping Lin, Mel Campbell, Chin-Chen Pan, Shu-Hui Lee, Hsin-Chen Lee, Muh-Hwa Yang, Hsing-Jien Kung, Pei-Ching Chang

Figure S1

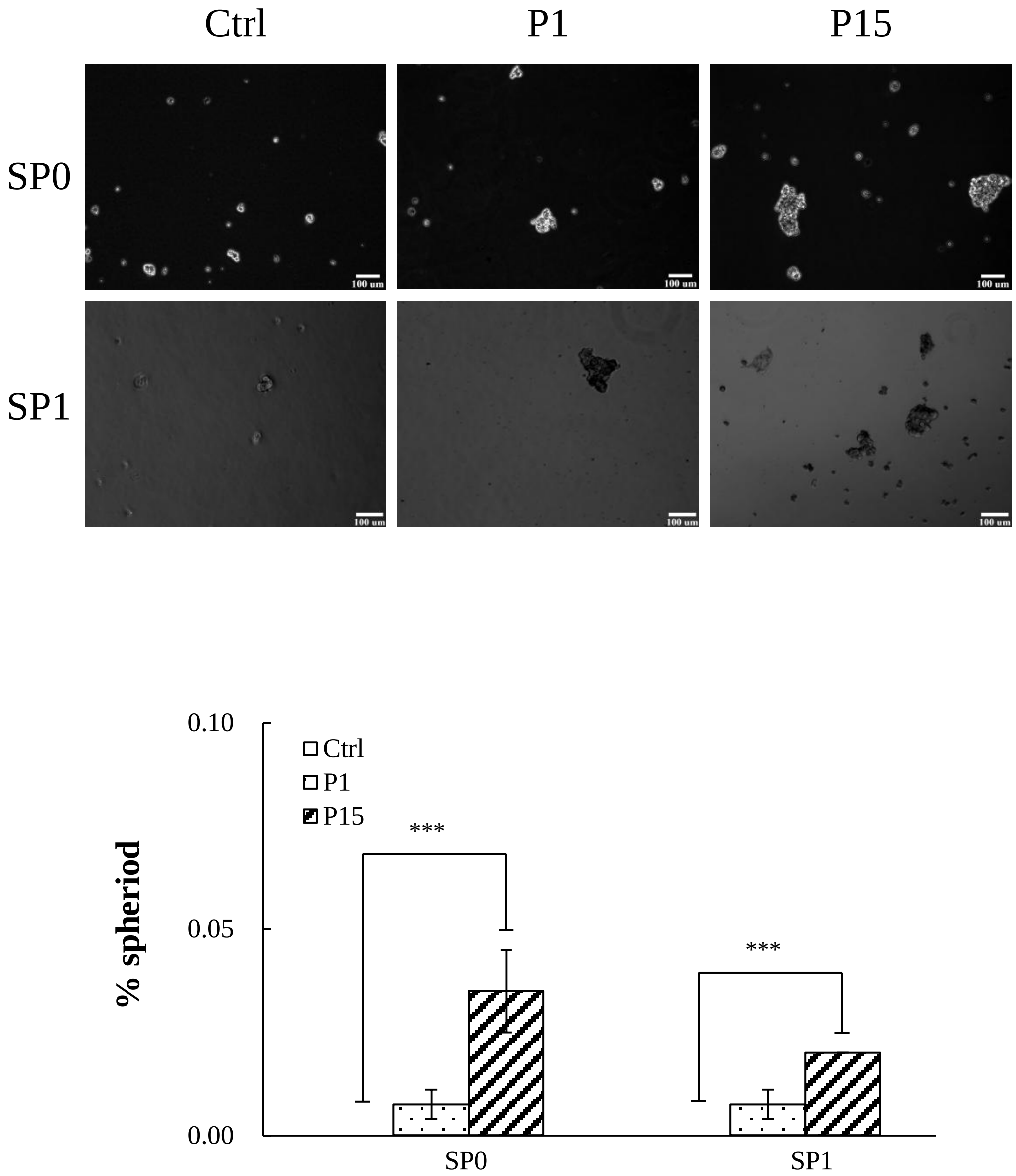
Ctrl
P1
P15
SP0
SP1
100 um
% spheriod
0.10
0.05
0.00
Ctrl
P1
P15
***
***
SP0
SP1

Figure S2

A

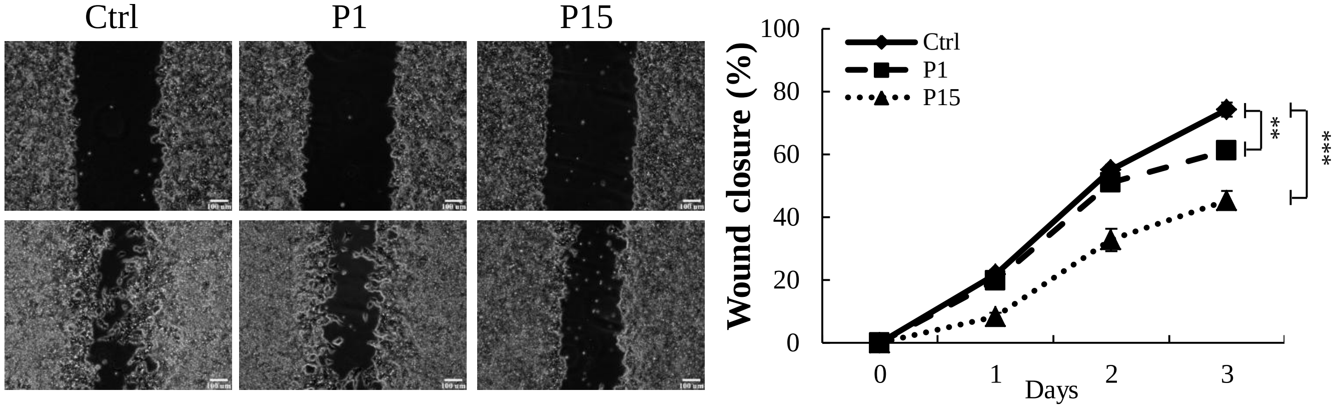

B

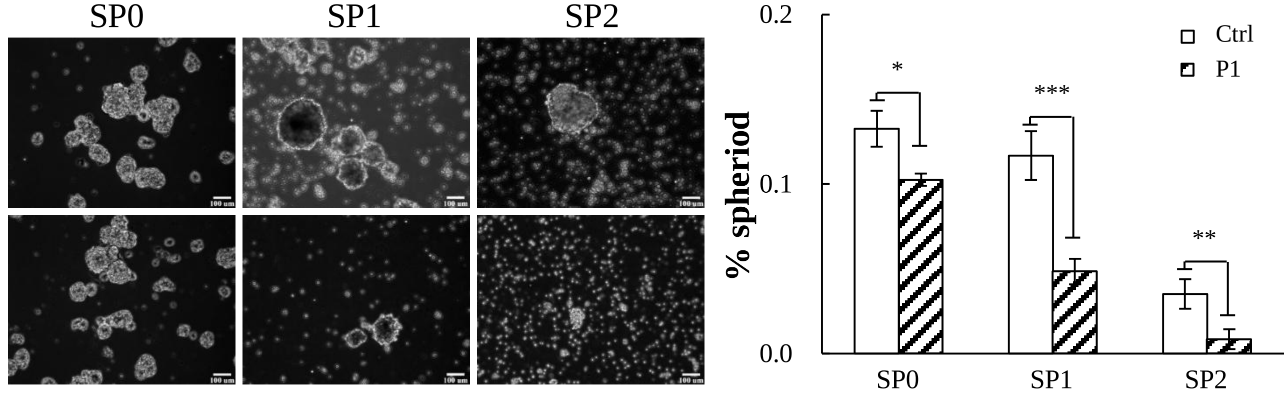

C

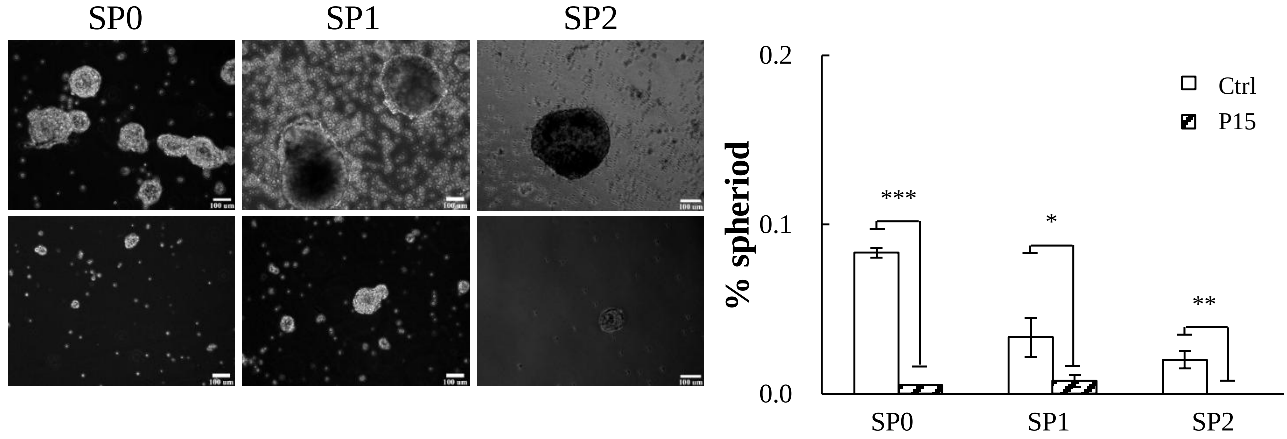

Figure S3

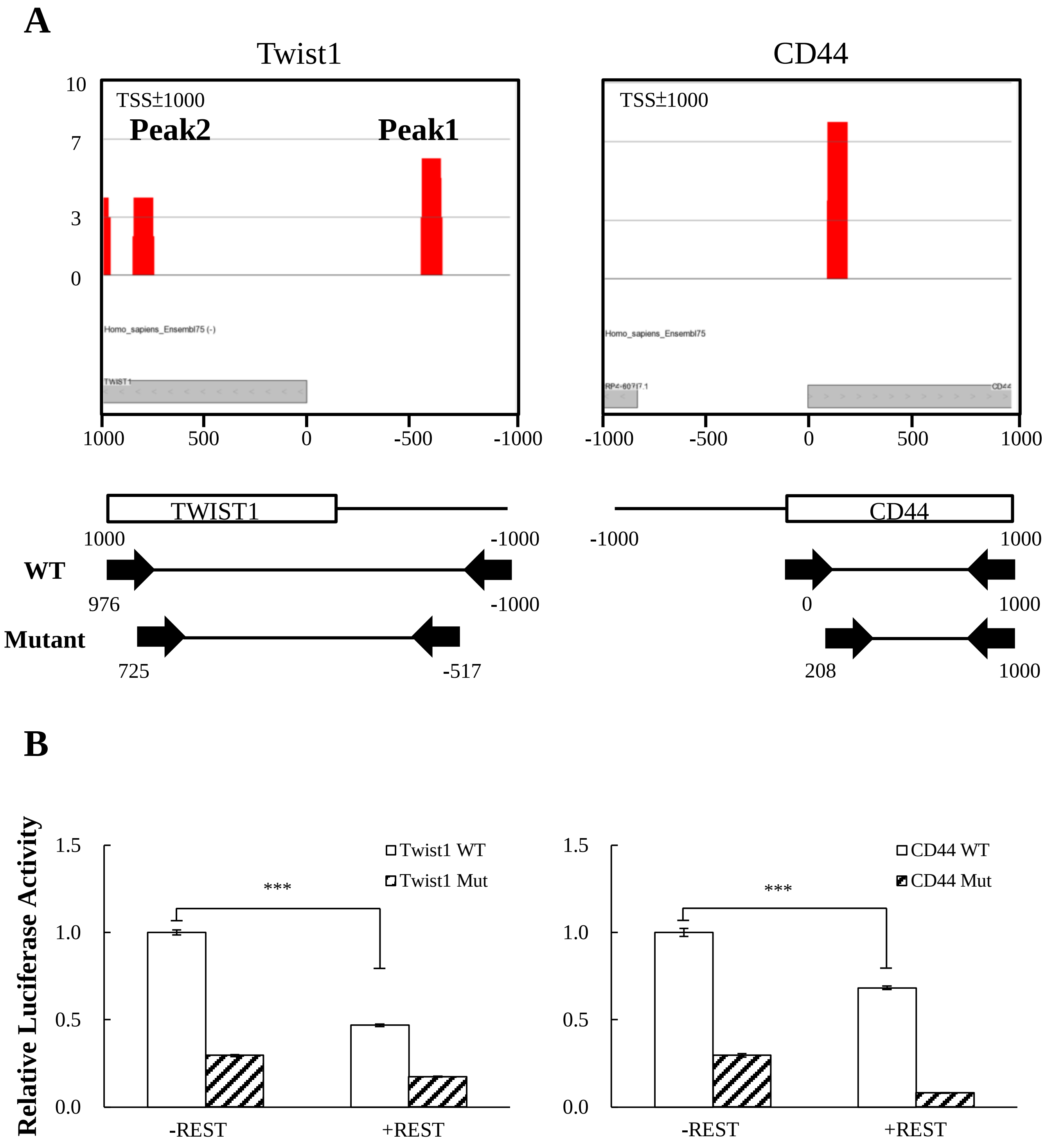
A
Twist1
CD44
TSS±1000
Peak2
Peak1
10
7
3
0
Homo_sapiens_Ensembl75 (-)
Homo_sapiens_Ensembl75
TWIST1
CD44
1000
500
0
-500
-1000
TWIST1
CD44
WT
Mutant
976
725
-517
208
B
Relative Luciferase Activity
1.5
1.0
0.5
0.0
Twist1 WT
Twist1 Mut
CD44 WT
CD44 Mut
***
-REST
+REST

Figure S4

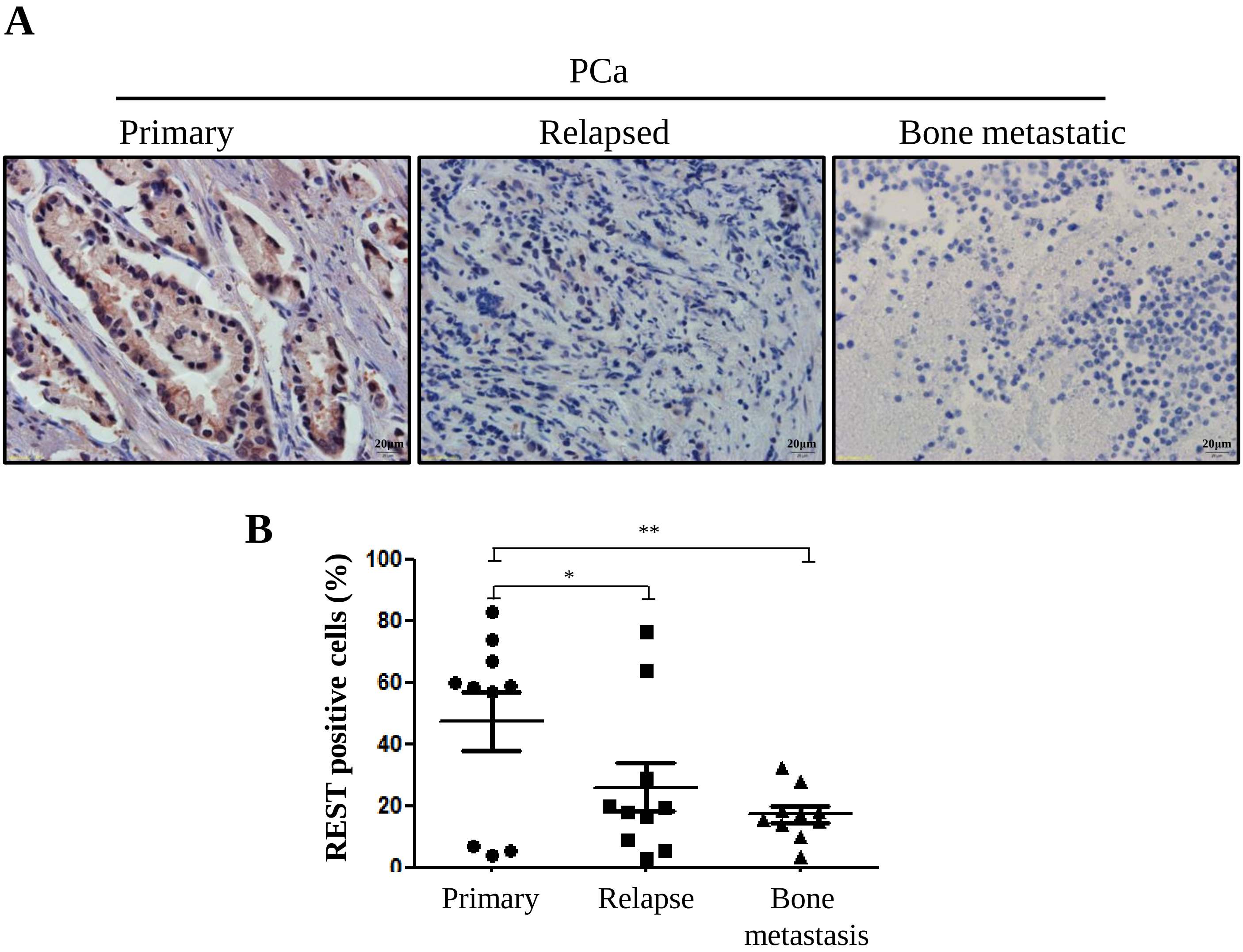
A
PCa
Primary
Relapsed
Bone metastatic
20μm
20μm
20μm
B
**
*
REST positive cells (%)
100
80
60
40
20
0
Primary
Relapse
Bone
metastasis

Figure S5

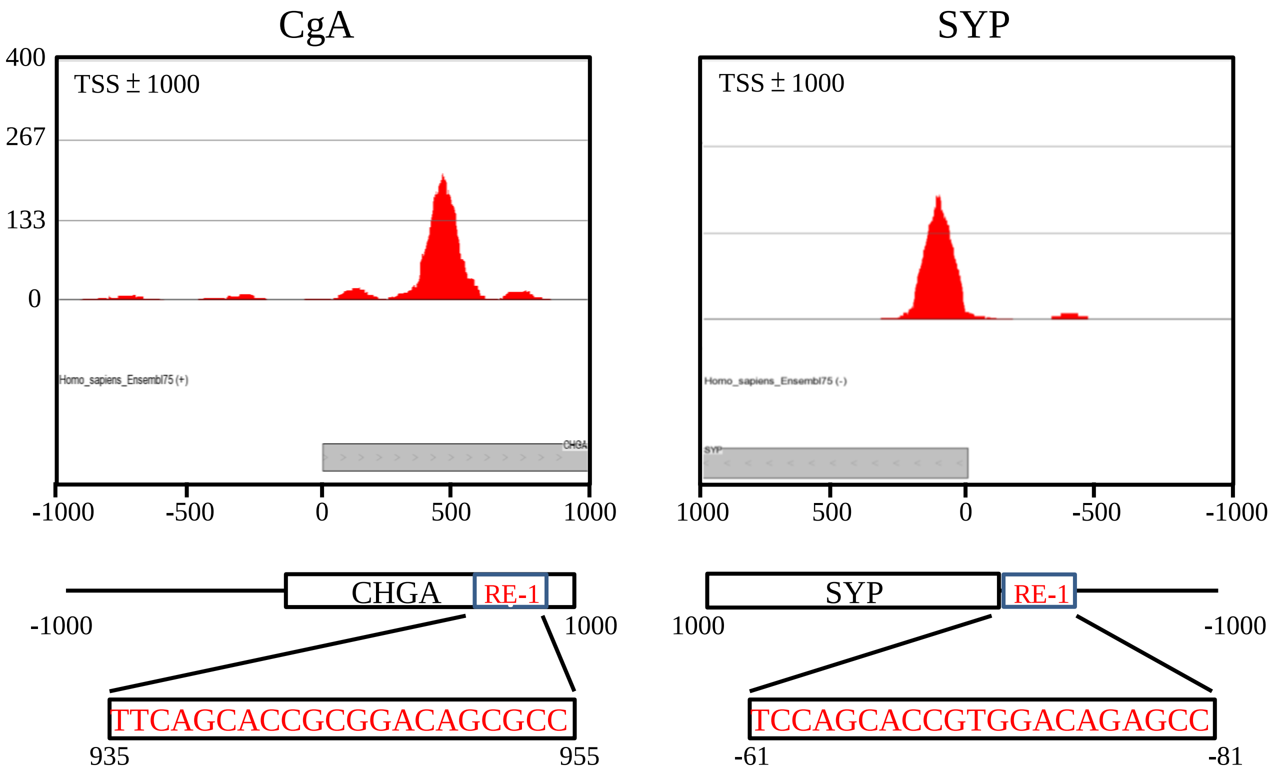

CgA
SYP
TSS ± 1000
TSS ± 1000
400
267
133
0
Homo_sapiens_Ensembl75 (+)
Homo_sapiens_Ensembl75 (-)
CHGA
SYP
-1000
-500
0
500
1000
1000
500
0
-500
-1000
CHGA
RE-1
SYP
RE-1
-1000
1000
1000
-1000
TTCAGCACCGCGGACAGCGCC
TCCAGCACCGTGGACAGAGCC
935
955
-61
-81

Figure S1. Long-term REST knockdown promotes stemness properties of PCa LNCaP cells. LNCaP-TR-shREST cells treated as described in Fig. 4D (SP0) were centrifuged and re-suspended in serum-free sphere medium for another 14 days (SP1). n=3. Data represent means ± SD. ***p<0.001 by *Student's-t* test.

Figure S2. Short-term and long-term overexpression of REST inhibits cell migration and stemness properties of CWR22Rv1 cells. (A) CWR22Rv1-TR-REST cells treated as described in Fig. 5B and 6B were subjected to wound healing assay. Images of cells were captured for continuous 3 days. Representative images are day 0 and 3 (left panel). The percentage of cell migration was calculated using three individual experiments (right panel). (B and C) CWR22Rv1-TR-REST cells treated as described in Fig. 5E (B) and 6E (C) (SP0) were centrifuged and re-suspended in serum-free sphere medium and cultured for 7 days. After 7 days, the re-suspension process was repeated and the cells were cultured for an additional 7 days. SP1 (serial passage 1, 14 day culture); SP2 (serial passage 2, 21 day culture). n=2. Data represent means ± SD. *p <0.05, ** p <0.01, ***p<0.001 by *Student's-t* test.

Figure S3. REST binding site(s) are essential for REST-mediated inhibition of Twist1 and CD44 promoter activities. (A) Potential RE-1 sites for REST binding in the promoter regions of Twist1 and CD44. ChIP-seq analysis shows REST enrichment on promoter region of the two genes (Upper panels). A schematic of reporter constructs of Twist1 and CD44 promoters with (WT) or without (Mutant) REST binding site(s) observed by ChIP-seq. (B) Reporter constructs were transfected with Renilla luciferase (8:1) in the presence or absence of REST. The reporter plasmids containing promoter harboring REST binding site(s) was arbitrarily set to 1 and the reporter activity of the REST binding site(s) deficient mutants was expressed as relative value. n=3. Data represent means ± SD. ***p<0.001 by *Student's-t* test.

Figure S4. Analysis of human primary, relapsed and bone metastatic PCa specimens for the expression of REST. (A) IHC staining was performed on ten primary, ten relapsed and ten bone metastatic PCa specimens using a specific antibody against REST. Representative images are shown. (B) Quantification data are the average with S.D. from 10 specimens in each group. *p<0.05, **p<0.01 by *Student's-t* test.

Figure S5. Potential RE-1 sites for REST binding in the promoter regions of CgA and SYP. ChIP-seq analysis shows REST enrichment on the promoter region of the

two genes. *In silico* analysis of the promoters (1 kb up- and down-stream of transcription start site (TSS)) of CgA and SYP by JASPAR revealed potential RE-1 site.

Table S1

Primers	
CgA_RT-qPCR_F	GGA TAC CGA GGT GAT GAA ATG C
CgA_RT-qPCR_R	CTG TGT TTC TTC TGC TGA TGT G
SYP_RTqPCR_F	GCC CAT GCT GGA CTT TCT G
SYP_RTqPCR_R	AGC CTG TCT CCT TAA ACA CGA
Twist 1_RT-qPCR_F	GAG TCC GCA GTC TTA CGA G
Twist 1_RT-qPCR_R	TGA ATC TTG CTC AGC TTG TC
N-cadherin_RT-qPCR_F	CCT CCA ATC AAC TTG CCA G
N-cadherin_RT-qPCR_R	ATG TGC CCT CAA ATG AAA CC
ZO-1_RT-qPCR_F	TCT CTT CCA GAA CCA AAG CC
ZO-1_RT-qPCR_R	TCA CCT TCC TCT AAG CCT TCC
CD44_RT-qPCR_F	CCA CAT TCT ACA AGC ACA ATC CA
CD44_RT-qPCR_R	AGT CCA TAT CCA TCC TTC TTC CT
Twist 1_Wang_RT-qPCR_F	GGA CAA GCT GAG CAA GAT TCA GA
Twist 1_Wang_RT-qPCR_R	TCT GGA GGA CCT GGT AGA GGA A
CD44_ChIP-qPCR_F	CGA TTA TTT ACA GCC TCA GCA
CD44_ChIP-qPCR_R	ACA GTG ACC TAA GAC GGA G
Twist1_ChIP-qPCR_peak1_F	ACT GGG TCG TTG TAG AGG G
Twist1_ChIP-qPCR_peak1_R	CCT GGG CGT TTC TGA AGA C
Twist1_ChIP-qPCR_peak2_F	CAA GCT GAG CAA GAT TCA GAC
Twist1_ChIP-qPCR_peak2_R	CTC GTG AGC CAC ATA GCT G
